# Advances in Nanotechnology-Based Cisplatin Delivery for ORL Cancers: A Comprehensive Review

**DOI:** 10.3390/ijms26115261

**Published:** 2025-05-30

**Authors:** Anda Ioana Morgovan, Eugen Radu Boia, Alexandru Catalin Motofelea, Alexandru Orasan, Mihaela Cristina Negru, Kristine Guran, Diana Maria Para, Daniela Sandu, Sonja Ciocani, Adrian Mihail Sitaru, Nicolae Constantin Balica

**Affiliations:** 1 Department of Ear, Nose and Throat, Faculty of Medicine, “Victor Babeș” University of Medicine and Pharmacy Timisoara, 2 Eftimie Murgu Sq., 300041 Timisoara, Romania; anda.morgovan@umft.ro (A.I.M.); alexandru.orasan@umft.ro (A.O.); mihaelaprodea@umft.ro (M.C.N.); guran.kristine@umft.ro (K.G.); diana.para@umft.ro (D.M.P.); ciocani_s@yahoo.com (S.C.); balica@umft.ro (N.C.B.); 2 Center for Molecular Research in Nephrology and Vascular Disease, Faculty of Medicine, “Victor Babeș” University of Medicine and Pharmacy, 300041 Timisoara, Romania; alexandru.motofelea@umft.ro; 3 OncoHelp Cancer Centre, Radiation Oncology Department, “Victor Babeș” University of Medicine and Pharmacy, Str. Rusu Sireanu nr. 34 Timisoara, 300041 Timisoara, Romania; daniela.sandu@umft.ro; 4 Department of Pediatric Surgery, “Louis Turcanu” Emergency Clinical Hospital for Children, Iosif Nemoianu Street 2, 300011 Timisoara, Romania; adrian.sitaru@umft.ro

**Keywords:** ORL cancers, cisplatin, nanocarriers, biotechnology

## Abstract

Otorhinolaryngological (ORL) cancers, including malignancies of the oral cavity, pharynx, and larynx, show significant challenges in oncology. Cisplatin, a platinum-based chemotherapy drug, remains a cornerstone of treatment but is often limited by systemic toxicity and resistance. A comprehensive literature review was conducted using recent studies and clinical trials focused on nanotechnology-based cisplatin delivery systems. The analysis covered various types of nanocarriers, their mechanisms, and advantages. Additionally, the limitations of nanotechnology-based cisplatin delivery systems were discussed. Findings indicate that lipid-based nanoparticles, polymeric nanoparticles, inorganic nanoparticles, and extracellular vesicles have demonstrated improved drug targeting, bioavailability, and reduced systemic toxicity in preclinical and clinical studies. Nanocarriers also offer potential for overcoming drug resistance and enabling combination therapy. However, challenges related to biocompatibility, scalability, and regulatory approval remain significant barriers to widespread clinical adoption. Nanotechnology offers a novel and promising approach to optimizing cisplatin delivery for ORL cancers. While preclinical studies demonstrate significant potential, further research and clinical validation are essential to translate these advancements into routine clinical practice. Addressing manufacturing and regulatory challenges will be critical for future research.

## 1. Introduction

Otorhinolaryngological (ORL) cancers, which include malignancies of the oral cavity, pharynx, and larynx, account for a significant proportion of global cancer cases. Approximately 7.7% of new cases of cancer diagnosed in 2020 were ORL cancers, with esophagus cancer having the highest incidence (3.1%) and also the highest death rate (5.5%) [1]. These cancers are often associated with risk factors such as tobacco use, alcohol consumption, human papillomavirus (HPV) infection, and environmental pollutants [2]. Despite advances in early detection and therapeutic strategies, ORL cancers continue to present high morbidity and mortality rates, particularly in advanced stages where treatment options become more limited.

The tumor microenvironment influences cancer progression and therapeutic responses [3]. Three critical factors that significantly affect tumor behavior and drug delivery are stromal density [4], hypoxia [5], and immune cell infiltration [6]. The interrelationships between these factors are essential for elucidating the complexity of tumor biology and improving treatment efficacy [7].

Stromal density—including different cell types, such as cancer-associated fibroblasts (CAFs) and immune cells—contributes to tumor progression and therapy resistance. Increased stroma can lead to alterations in the extracellular matrix, impacting drug diffusion and efficacy. The presence of CAFs has been associated with a more aggressive tumor phenotype. Tumor-associated macrophages (TAMs) can constitute a significant component of the tumor stroma and have been shown to contribute to both local tumor proliferation and systemic metastasis, particularly in glioblastoma [8,9]. Furthermore, the density of immune infiltrates is correlated with prognosis, indicating that manipulating stromal components may enhance therapeutic outcomes [10,11].

Hypoxia, a common feature in solid tumors, influences tumor behavior by promoting angiogenesis, immune evasion, and drug resistance. The hypoxic microenvironment can trigger adaptive responses, leading to the upregulation of escape mechanisms, such as the expression of immune checkpoints, such as PD-L1, on immune cells like macrophages and myeloid-derived suppressor cells (MDSCs), facilitating immune tolerance [12,13]. Furthermore, hypoxia is linked to metabolic changes that promote tumor cell survival and increase local immunosuppression, decreasing T cell activity and promoting a state of exhaustion that significantly reduces the efficacy of various therapeutic strategies [12,14].

The dynamic interplay between hypoxia and immune cell infiltration further complicates therapeutic strategies. Targeting pathways involved in hypoxia can enhance T cell infiltration and boost immunotherapy efficacy, particularly in tumors that traditionally show poor immune responses [15,16]. Studies show that reducing hypoxic stress has been shown to restore immune cell function and improve outcomes in conditions such as melanoma and bladder cancer [16,17].

Cisplatin, a platinum-based chemotherapeutic agent, remains one of the most effective drugs for treating ORL cancers [18,19]. It functions primarily by forming DNA adducts, which interfere with DNA replication and transcription, ultimately leading to apoptosis in rapidly dividing cancer cells [20,21]. However, despite its efficacy, cisplatin is associated with several drawbacks. Its nonspecific distribution results in severe systemic toxicities [22], including nephrotoxicity [23], ototoxicity [24], neurotoxicity [25], and myelosuppression [26]. These adverse effects often limit the maximum tolerable dose and lead to dose reductions or treatment discontinuation, negatively impacting therapeutic outcomes.

Another significant challenge in cisplatin-based chemotherapy is the development of resistance. Tumor cells develop various mechanisms to escape cisplatin-induced cytotoxicity, such as enhanced DNA repair mechanisms, increased drug efflux, and inhibition of apoptotic pathways [10,27,28]. These resistance mechanisms significantly reduce cisplatin’s effectiveness and contribute to treatment failure in ORL cancers. Consequently, improving cisplatin delivery while mitigating resistance and toxicity has become a major focus of oncological research.

Nanotechnology has emerged as a promising approach to addressing these challenges. By engineering nanoparticles as drug carriers, researchers have explored methods to improve cisplatin delivery, increase tumor specificity, and reduce systemic toxicity. Various nanoparticle-based delivery systems, including lipid-based, polymeric, inorganic, and biological nanocarriers, have demonstrated significant potential in preclinical and early clinical studies. These nanocarriers offer advantages such as controlled drug release, enhanced tumor penetration, and the ability to bypass resistance mechanisms, making them attractive candidates for optimizing ORL cancer therapy [29,30,31].

This review aims to provide a comprehensive analysis of the advancements in nanotechnology-based cisplatin delivery for ORL cancers. It will discuss the mechanisms of cisplatin action and resistance, different nanocarrier systems, their advantages and challenges, and the current landscape of preclinical and clinical studies. By highlighting the potential of nanotechnology in improving ORL cancer treatment, this review seeks to pave the way for future research and clinical translation of these innovative approaches.

## 2. Materials and Methods

This narrative review was designed to summarize and interpret recent advances in nanotechnology-based cisplatin delivery for ORL cancers, emphasizing depth of discussion over exhaustive coverage. Between January and February 2025, we performed targeted searches in PubMed, Scopus, and Google Scholar using combinations of the terms “cisplatin”, “nanocarriers”, “nanotechnology”, “lipid-based nanoparticles”, “polymeric nanoparticles”, “extracellular vesicles”, and “head and neck cancer”. To maintain focus, Google Scholar screening was limited to the first 200 results per keyword combination. We included peer-reviewed original research articles, early-phase clinical trials, and selected reviews published in English that provided quantitative data on drug delivery mechanisms, therapeutic efficacy, toxicity reduction, or resistance modulation in ORL cancer models. Editorials, letters, conference abstracts, non-English papers, and studies lacking substantive delivery- or outcome-related data were excluded. Titles and abstracts were first screened for relevance, followed by full-text review of articles meeting these criteria. The final selection was organized by nanocarrier class (lipid-based, polymeric, inorganic, biological), and within each class, studies were compared narratively in terms of targeting strategy (passive vs. active), in vitro and in vivo efficacy, immunological effects, and translational stage.

## 3. Mechanisms of Cisplatin Action and Resistance

**Figure 1:**
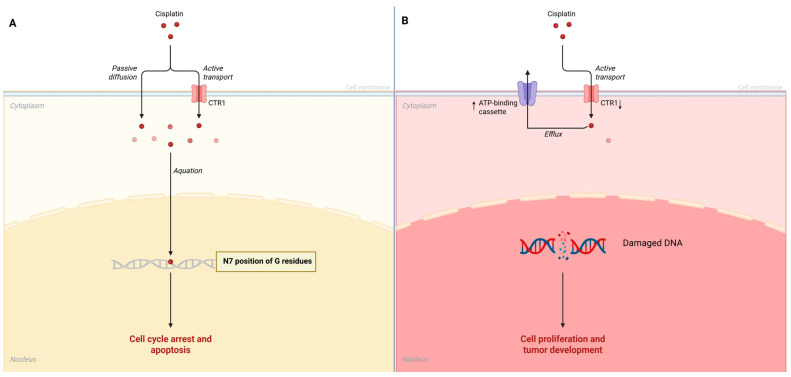
(**A**) Cisplatin mechanism of action. (**B**) Cisplatin resistance mechanisms in cancer cells.

Cisplatin is a platinum-based chemotherapeutic agent widely used to treat various cancers, including those of the oral and oropharyngeal regions, due to its efficacy in inducing DNA damage leading to apoptosis in malignant cells. The primary mechanism of action involves the formation of DNA cross-links, particularly at the N7 position of guanine residues, which disrupts DNA replication and transcription, triggering cellular signaling pathways that lead to programmed cell death [32]. However, cancer cells often develop resistance to cisplatin, complicating treatment regimens and leading to poor clinical outcomes. Understanding the mechanisms underlying cisplatin resistance is crucial for improving therapeutic strategies and outcomes in ORL cancers. The mechanisms of cisplatin resistance can be broadly categorized into several molecular mechanisms. These include alterations in drug uptake and efflux, enhanced DNA repair processes, and changes in apoptosis signaling pathways. Additionally, genetic and epigenetic changes in cancer cells can establish new resistance mechanisms. Mutations and alterations in gene expression profiles may decrease cisplatin-related damage responses [33]. Overall, the interplay of these mechanisms signifies a complex landscape of cisplatin resistance within ORL cancers, necessitating the exploration of combination therapies to enhance treatment efficacy. For instance, the copper transporter CTR1 has been identified as a critical factor in mediating cisplatin uptake (Figure 1A); reduced expression of CTR1 has been associated with decreased drug accumulation in cells, contributing significantly to resistance (Figure 1B) [34]. Additionally, increased expression of ATP-binding cassette (ABC) transporters facilitates the efflux of cisplatin from cancer cells, which also diminishes its intracellular concentration and effectiveness (Figure 1B) [33]. Furthermore, enhanced DNA repair capabilities, particularly through nucleotide excision repair pathways, enable cancer cells to overcome cisplatin-induced damage [33]. Another significant factor contributing to resistance is the role of glutathione and reactive oxygen species (ROS) in modulating cellular responses to cisplatin. High levels of glutathione can neutralize the oxidative stress induced by cisplatin, thereby protecting cancer cells and increasing their chance of survival [35]. In ORL cancers, studies have elucidated the production of ROS as a response to cisplatin treatment, suggesting that the modulation of oxidative stress could serve as a potential therapeutic target to enhance cisplatin’s effectiveness against resistant cancers [36]. Importantly, recent investigations have highlighted the influence of microRNAs (miRNAs) in modulating cisplatin resistance. Specific miRNAs can regulate the expression of genes involved in drug resistance pathways, facilitating the survival of cancer cells in the presence of cisplatin [37]. For example, miR-643 was shown to be transferred from cisplatin-resistant cells to sensitive ones, imparting resistance properties and complicating treatment options further [37].

## 4. Nanotechnology in Cisplatin Delivery

The deployment of nanotechnology in the delivery of cisplatin for treating oral and laryngeal cancers has attracted significant interest due to its potential to enhance drug efficacy and overcome various forms of drug resistance often associated with these malignancies. Nanoparticle-based drug delivery systems, particularly for cisplatin, have gained considerable attention in cancer therapeutics due to their potential to enhance targeting efficiency and reduce systemic toxicity. The strategies employed in nanoparticle formulations broadly fall into two categories: passive targeting and active targeting, each with distinct mechanisms and implications for internalization pathways. Passive targeting relies on the enhanced permeability and retention (EPR) effect, a phenomenon observed in tumor vasculature, which allows nanoparticles to accumulate preferentially in tumor tissues. The leaky blood vessels and compromised lymphatic drainage mechanisms common in tumors facilitate the extravasation of nanoparticles from the bloodstream into the tumor microenvironment [38]. This method benefits from the natural tendency of nanocarriers to passively accumulate in neoplastic tissues based on size and surface characteristics without the need for specific targeting ligands. Focusing on cisplatin, studies have demonstrated that encapsulating cisplatin within polymeric nanoparticles can enhance its delivery and lower its nephrotoxicity, a significant side effect of conventional cisplatin therapy [39]. In contrast, active targeting involves the functionalization of nanoparticles with specific ligands that bind to overexpressed receptors on tumor cells. This method enhances specificity in drug delivery, minimizing effects on healthy tissues while maximizing therapeutic efficacy [40]. Active targeting can dramatically increase cellular internalization rates, as targeting ligands facilitate receptor-mediated endocytosis pathways, thereby promoting efficient uptake of the drug within cancer cells.

The internalization pathways in active targeting often involve clathrin-mediated endocytosis or caveolae-mediated endocytosis, depending on the specific receptor–ligand interactions initiated by the nanoparticles. This enhanced internalization can facilitate a more efficient drug release profile, ensuring therapeutic concentrations of cisplatin are achieved within the target cells [41].

The internalization of nanoparticles can significantly influence their therapeutic outcome. For passively targeted nanoparticles, internalization occurs primarily through pinocytosis or diffusion, driven by nanoparticle size, shape, and surface charge [42]. However, for actively targeted nanoparticles, specific endocytosis pathways play a critical role.

By utilizing nanocarriers, researchers aim to enhance the targeted delivery of cisplatin, minimize its adverse effects, and improve therapeutic outcomes for patients with ORL cancers. Nanoparticles offer a versatile platform for drug delivery, enabling the encapsulation of cisplatin within various matrices including liposomes, polymeric nanoparticles, and metal-based nanoparticles. For instance, studies have shown that liposomal nanoparticles containing cisplatin exhibit enhanced cytotoxic activity compared to free cisplatin, significantly improving the drug’s efficacy against various cancers.

**Figure 2:**
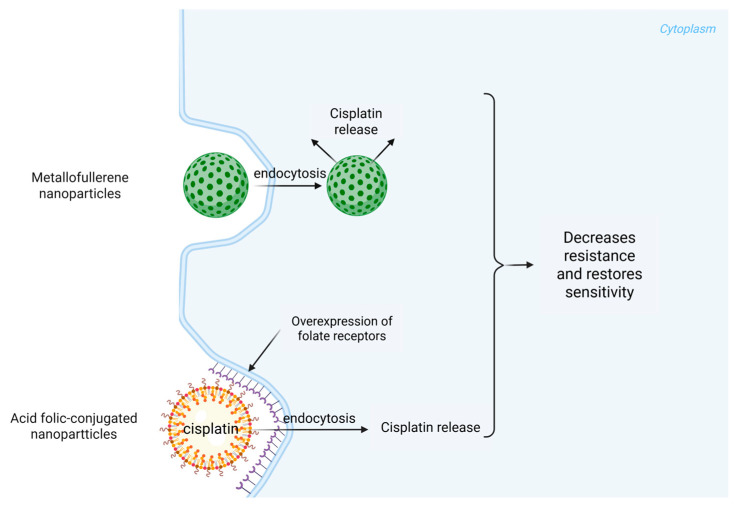
Cisplatin delivery routes with metallofullerene and acid folic-conjugated nanoparticles.

The lipid-based formulations improve the bioavailability of the drug by facilitating its entry into tumor cells while simultaneously reducing exposure to non-target tissues [43]. Furthermore, cisplatin-loaded methoxy-poly(ethylene glycol)-block-poly(L-glutamic acid) nanoparticles have displayed promising delivery characteristics, prompting enhanced antitumor effects due to their ability to modulate the intracellular release conditions of the drug [44]. A key advancement in nanotechnology relates to its ability to overcome multidrug resistance (MDR) mechanisms that tumor cells employ to evade chemotherapeutic agents like cisplatin. Recent findings suggest that metallofullerene nanoparticles may reactivate endocytosis pathways in resistant cancer cells, allowing for higher intracellular concentrations of cisplatin (Figure 2) [45]. Incorporating such mechanisms into the design of cisplatin delivery systems can potentially reverse resistance and restore sensitivity in tumors that have begun to lose responsiveness to standard therapies. To create a cisplatin delivery system for ORL cancers, the use of folic acid-conjugated nanoparticles has emerged as a promising strategy. These nanoparticles utilize the overexpression of folate receptors in certain tumor types to enhance cellular uptake, thereby increasing the efficacy of cisplatin while minimizing off-target effects (Figure 2) [46].

Biodegradable polymeric nanoparticles allow for a controlled release of the drug, facilitating sustained therapeutic concentrations within the tumor microenvironment, a factor critical for achieving optimum treatment efficacy in ORL cancers [47]. This strategic release profile aims not only to maintain drug action over an extended period but also to reduce normal tissue toxicity by preventing high plasma concentration peaks that are typical with conventional drug administration methods. Furthermore, combining cisplatin with other therapeutic agents delivered via nanoparticles has shown synergistic effects against resistant cancer phenotypes. Studies have noted that co-delivering cisplatin with PARP (Poly (ADP-ribose) polymerase) inhibitors in layered nanoparticles can enhance therapeutic outcomes in ovarian cancer models, a strategy that may likewise be applicable in ORL cancer settings [48].

Nanoparticle-mediated delivery of cisplatin presents a promising avenue to enhance immune activation alongside chemotherapy. Three main strategies appear to dominate the current literature: the use of immunostimulatory agents, enhancing immune cell infiltration, and the development of synergistic treatment combinations.

One innovative approach involves the use of immunostimulatory nanoparticles, particularly those that combine cisplatin with immune adjuvants. For instance, Hernández-Gil et al. developed iron oxide nanoparticles loaded with a Pt(IV) prodrug of cisplatin and polyinosinic-polycytidylic acid, a double-stranded RNA (dsRNA) that functions as an immunomodulator. This combination improves the cytotoxic effects of the cisplatin prodrug and enhances the innate immune response, promoting a more effective antitumor environment [49]. This aligns with findings from other studies indicating that adding immune-modulating factors to theranostic nanoparticles can boost the infiltration of T and B lymphocytes within tumors, thereby heightening the potential for an effective immune response [50].

Enhancing immune cell infiltration is crucial for effective tumor control. Nanoparticles can be designed to actively target the tumor microenvironment, promoting the accumulation of cytotoxic T cells and other immune effectors. Hoffmann et al. describe therapies utilizing nanoparticles for head and neck cancers, where cisplatin delivery is paired with immune activators, leading to enhanced immune cell presence and improved therapeutic outcomes [51]. Similarly, the design of transformable nanoparticles that elicit immunogenic cell death through reactive oxygen species (ROS) generation—alongside cisplatin—demonstrates a synergistic approach to boosting both direct tumor killing and immune activation [52].

Another strategy described in the literature is targeting cisplatin with agents that modulate the immune microenvironment. Cytosolic DNA sensing, the cyclic GMP-AMP synthase-stimulator of interferon genes (cGAS-STING) signaling pathway, has been identified as a crucial mediator of immune responses to cisplatin, as it not only inhibits cancer cell proliferation but also enhances the infiltration of CD8+ T cells and dendritic cells in tumor settings [53,54]. Utilizing nanoparticles loaded with cisplatin and designed to activate this pathway could result in superior anticancer efficacy due to the dual action on tumor cells and the immune system alike [55].

Furthermore, combining cisplatin with other immunotherapeutic agents may provide additive or synergistic effects. Co-delivery systems combining cisplatin with checkpoint inhibitors or additional chemotherapeutics have shown promise in overcoming resistance mechanisms associated with cisplatin therapy [56,57]. Such approaches are expected to create a more favorable tumor microenvironment for immune activation and decrease the chances of tumor recurrence.

Moreover, the integration of RNA interference (RNAi) techniques with cisplatin nanocarriers has successfully silenced genes responsible for drug resistance, presenting yet another layer of complexity and effectiveness in overcoming therapeutic barriers in cancer treatment [58,59,60]. Nanotechnology also allows for the engineering of smart drug delivery systems that respond to environmental stimuli. For instance, redox-responsive mesoporous silica nanoparticles can release cisplatin more effectively in the reductive environment typically found in tumor tissues, thereby enhancing the targeted action of the drug [61,62]. Such systems reflect a significant advancement in the precision of chemotherapeutic delivery, positioning nanotechnology as a cornerstone of future cancer therapies. The current research highlights the critical role of nanotechnology in the transformation of cisplatin delivery systems for ORL cancers. With ongoing studies aimed at refining the properties and functionalities of nanocarriers, the focus remains on achieving an optimum balance of efficacy, safety, and patient quality of life. As nanotechnology continues to advance, the prospects for improved treatment regimens utilizing cisplatin grow more promising, potentially setting new standards in cancer therapeutics (Figure 2).

## 5. Current Clinical and Preclinical Studies

The application of nanotechnology for the delivery of cisplatin in oral and oropharyngeal cancers represents a significant advancement in therapeutic strategies aimed at improving the efficacy and reducing the adverse effects of this important anticancer drug. Recent clinical and preclinical studies have underscored the role that nanoparticle formulations can play in enhancing the pharmacokinetics of cisplatin, thereby improving its tumor-targeting capabilities while minimizing systemic toxicity (Table 1).

Research indicates that nanoparticles can significantly enhance drug solubility and stability, which are often limitations with conventional cisplatin formulations. For instance, studies have demonstrated that nanoliposomes encapsulating cisplatin improve tumor targeting and enhance drug penetration into cancer cells, effectively addressing issues related to MDR [63,64]. Such formulations can facilitate intracellular delivery by exploiting the enhanced permeability and retention (EPR) effect, resulting in more effective cisplatin delivery directly to the tumor site [36,65]. These strategies can potentially reduce the required dosage of cisplatin, thereby decreasing side effects associated with higher systemic exposure.

Furthermore, targeting drug delivery systems that utilize surface modifications of nanoparticles can tailor the release profile of cisplatin. Studies have shown that co-encapsulation systems, which combine cisplatin with other therapeutic agents, can enhance chemotherapeutic efficacy through synergistic mechanisms. For example, one study highlighted the benefits of co-delivering cisplatin with agents that reverse MDR, such as paclitaxel, through a targeted nanosystem which resulted in increased apoptosis of resistant cancer cells [31]. The use of biodegradable polymeric nanoparticles also supports sustained drug release, thereby prolonging therapeutic effects and minimizing toxic peaks commonly associated with traditional administration routes [43].

Another critical aspect of using nanotechnology in cisplatin therapy for ORL cancers is its potential to modulate cellular pathways that increases the resistance. Targeting signaling pathways associated with apoptosis can enhance the sensitivity of cancer cells to cisplatin. Research indicates that inhibiting or downregulating proteins involved in the apoptotic process, such as SIRT2, can contribute to a reversion of resistance in ovarian cancer cells, thereby allowing cisplatin to exert its cytotoxic effects more effectively [10,35]. Advances in understanding tumor biology also facilitate the creation of nanoparticles that can specifically target cancerous tissues, guided by tumor-specific antigens or receptors, further improving treatment selectivity and efficacy [47,66].

The incorporation of natural compounds within nanocarriers is an emerging area of interest, as such combinations can decrease the nephrotoxic effects frequently associated with cisplatin therapy. For example, honokiol, which has been shown to possess protective effects against cisplatin-induced toxicity, can be delivered using nanoparticles to enhance both its bioavailability and chemosensitivity in treated cancer cells [67,68]. This dual action not only preserves the therapeutic benefits of cisplatin but also addresses its deleterious side effects, signifying an important step forward in personalized cancer therapy. Moreover, antioxidant micronutrients such as beta-carotene serve as oral carcinogenesis inhibitors, and in synergy with vitamin E, vitamin C, and chemotherapeutic drugs, they not only induce clinical regression of oral leukoplakia but also significantly enhance the overall efficacy of cancer therapy [69].

Preclinical investigations are focusing on various types of nanoparticles, including gold nanoparticles and mesoporous silica nanoparticles. These novel systems have been shown to not only deliver cisplatin but also allow for imaging and diagnostic functionalities [13,70]. Such multifaceted nanoparticles can be engineered to respond to specific physiological conditions, such as pH changes typical of the tumor microenvironment, ensuring that cisplatin is released precisely where it is needed most [62,71].

While several preclinical studies demonstrate promising outcomes in utilizing nanotechnology for cisplatin delivery, transitioning these findings into clinical practice remains a vital objective. The challenges surrounding this transition include rigorous evaluation of safety, long-term effects, and the complexity of manufacturing reproducibly. Ongoing trials and accumulating evidence from early-phase studies are gradually making a pathway toward the clinical implementation [61] of these innovative nanoparticle-based delivery systems [72].

**Table 1:** Advantages of nanotechnology-based delivery systems of cisplatin in ORL and other cancers.

Aspect	Nanotechnology-Based Approach	Nanocarrier Type	Study Model	Type of Cancer	Advantages	References
Drug Bioavailability and Pharmacokinetics	Nanoparticles encapsulating cisplatin	Lipid-based nanoparticles	In vitro, in vivo	ORL cancer, ovarian cancer	Protect cisplatin from degradation, prolong systemic circulation, and improve bioavailability at the tumor site, particularly in OSCC treatment.	[44,73,74]
Specificity and Targeting	Targeted delivery via functionalized nanoparticles (e.g., folic acid conjugation)	Polymeric particles, mesoporous silica particles	In vitro	Cervical cancer (HeLa cell line)	Enhanced drug delivery to cancer cells that overexpress receptors, improving efficacy while minimizing off-target effects and reducing systemic toxicity.	[46,75]
Overcoming Drug Resistance	Nanoparticle-based systems evading efflux pumps	Polymeric particles, mesoporous silica particles	In vitro	Oral cancer	Prevent drug resistance by facilitating drug accumulation inside cancer cells, bypassing efflux mechanisms like drug efflux pumps.	[29,43]
Controlled Release Systems	Redox-responsive mesoporous silica nanoparticles	Mesoporous silica nanoparticles	In vitro	Cervical cancer (HeLa cell line)	Release cisplatin in reducing environments of cancer cells, enhancing cytotoxicity against malignant cells while minimizing systemic exposure.	[46]
Biocompatibility and Pharmacokinetic Modulation	Custom nanoparticle systems (e.g., solid lipid nanoparticles)	Lipid nanoparticles, polymeric nanoparticles	In vitro	Various ORL cancer, ovarian cancer cell	Modify drug release profiles, enhance solubility and stability, and improve oral bioavailability, ensuring more efficient chemotherapy in oral cancer.	[47,74]
Combination Therapies	Combination of cisplatin with other therapeutic agents (antioxidants, immune modulators)	Organic nanoparticles, polymeric nanoparticles, lipid-based nanoparticles	In vitro, in vivo	Oral cancer, laryngeal cancers	Enhance overall cytotoxicity and counteract chemoresistance by integrating other therapeutic agents within nanoparticles, boosting the effectiveness of cisplatin.	[62,76,77]
Multifunctional Platforms for Diagnostics and Therapy	Nanoparticles integrating imaging agents	Gold nanoparticles, inorganic nanoparticles, polymeric nanoparticles	In vitro	Laryngeal cancer	Enable real-time monitoring of treatment efficacy, allowing adaptive strategies based on tumor response, optimizing treatment outcomes.	[43,78]
Stimuli-Responsive Release Mechanisms	Tumor-specific signal-responsive nanocarriers (e.g., pH-sensitive or enzyme-responsive systems)	Polymeric nanoparticles, drug-delivery platforms	In vitro	Various ORL cancers	Enable precise drug release within the tumor, enhancing therapeutic efficacy and minimizing systemic toxicity by evading healthy tissues.	[79]

As shown in Table 2, lipid-based nanoparticles lead the way in late-stage clinical development, whereas other platforms such as mesoporous silica and extracellular vesicles remain largely at the preclinical stage.

## 6. Challenges and Future Perspectives

While the integration of nanotechnology in cisplatin delivery shows significant promise for improving the treatment of ORL cancers, several challenges remain that need to be addressed before its widespread clinical adoption. One of the primary concerns is biocompatibility, which is the ability of the nanoparticle formulation to perform its intended function without showing significant adverse biological responses. Specifically, in the context of cisplatin-loaded nanoparticles, this includes minimizing cytotoxic effects on healthy tissues, avoiding undesired immune activation, and ensuring favorable pharmacokinetics and biodistribution [44]. Nanoparticles used for drug delivery must be safe for human use without inducing adverse immune responses or toxicity. The precise interaction of nanoparticles with biological tissues needs to be thoroughly understood to ensure they do not trigger inflammatory or allergic reactions [62].

Additionally, the scalability of nanoparticle manufacturing presents another challenge. Current production methods may not be suitable for large-scale, cost-effective manufacturing, which could hinder the widespread use of these treatments in clinical settings. Overcoming these manufacturing barriers will require the development of efficient, reproducible methods to produce nanoparticles in large quantities while maintaining their stability and therapeutic efficacy.

The clinical translation of nanoparticle systems for delivering cisplatin in ORL cancers is an area of growing interest, driven by the need to enhance therapeutic efficacy while minimizing systemic toxicity. Several nanocarrier approaches are currently being investigated in clinical settings, demonstrating promising developments.

Liposomal formulations of cisplatin, such as Lipoplatin, are being evaluated for their targeted delivery capabilities. A meta-analysis comparing liposomal cisplatin to conventional non-liposomal cisplatin in head and neck squamous cell carcinoma (HNSCC) suggests that the liposomal formulation may offer improved efficacy, although definitive conclusions regarding overall survival and progression-free survival are still needed [80]. Various clinical trials are ongoing that explore these formulations, highlighting the established safety and tolerability in patient populations [81,82].

In addition, polymeric systems, such as polysaccharide vesicles and PEGylated micelles, have shown potential in preclinical studies for their ability to co-deliver multiple anticancer agents alongside cisplatin. These formulations have been noted for their reduced toxicity profiles and improved pharmacodynamics, prompting future clinical evaluations [62,83]. Recent trials are focused on the use of hybrid nanocarriers based on poly lactic-co-glycolic acid (PLGA) and other approved polymers to deliver cisplatin in a targeted manner [84], with early results suggesting beneficial outcomes.

New strategies such as the integration of machine learning with molecular modeling are also being explored to improve the design and function of these nanocarriers, addressing challenges related to drug delivery efficiency and biological interactions. This innovative approach may pave the way for rationally designed nanocarrier systems that could integrate more effectively into the clinical setting [85].

While progress in the clinical translation of these nanoparticle formulations appears promising, it is crucial that ongoing and future trials evaluate their efficacy and safety in larger, diverse populations.

Future research should focus on optimizing nanoparticle formulations to improve their biocompatibility, drug release profiles, and targeting mechanisms.

Finally, while preclinical studies and early-phase trials have shown positive results, large-scale clinical trials are essential to establish the broader clinical applicability of nanotechnology-based cisplatin delivery systems. These trials will help determine the appropriate dosing, patient selection criteria, and long-term outcomes of this treatment approach, ultimately determining whether it can become a standard of care in the treatment of ORL cancers.

## 7. Conclusions

Nanotechnology represents a revolutionary approach that holds great promise for addressing the inherent limitations of traditional cisplatin therapy in the treatment of ORL cancers. By enhancing the precision, efficacy, and safety of cisplatin delivery, nanotechnology could significantly improve the outcomes for patients suffering from these aggressive cancers. However, to fully realize the potential of nanotechnology in clinical practice, further research and clinical validation are critical. Investigating more efficient nanoparticle formulations, conducting large-scale clinical trials, and navigating regulatory challenges will be essential steps in translating these technological advancements into routine clinical use. If successful, these innovations could usher in a new era of cancer treatment, improving survival rates and quality of life for patients with ORL cancers. 

## Figures and Tables

**Table 2:** Comparative features of major nanocarrier platforms for targeted cisplatin delivery.

Feature	Lipid-Based NPs	Polymeric NPs	Inorganic NPs	Mesoporous Silica NPs	Extracellular Vesicles	Hybrid/Composite NPs
Biocompatibility	Excellent; reduced nephrotoxicity	Generally good; potential polymer toxicity	Moderate; long-term metal accumulation concerns	Moderate; biodegradability issues	Intrinsic; immune evasive	Variable, depending on component materials
Drug-loading capacity	Limited	High	Moderate	Very high (large pore volume for co-loading)	Low yield; heterogeneous cargo	Tunable via choice of polymer/lipid/metal blends
Release profile	Passive EPR-mediated accumulation	Controlled/sustained	Multifunctional (imaging + therapy)	Stimuli responsive	Natural cargo delivery; endogenous release cues	Synergistic release modes (e.g., burst + sustained)
Circulation stability	Challenged (stability in blood)	Batch-to-batch variability	Very high	Fine surface engineering	Moderate; stability varies with isolation method	Depends on formulation and core–shell architecture
Manufacturing complexity	Moderate	Moderate	High	Moderate	High (low yield, scalability issues)	High (multiple components and processing steps)
Safety/toxicity concerns	Generally low systemic toxicity	Polymer-related toxicity possible	Metal accumulation; unclear long-term fate	Silicosis risk; slow biodegradation	Low immunogenicity; cargo heterogeneity risks	Regulatory pathway often unclear
Tumor-targeting specificity	Passive (EPR)—moderate; active with ligands possible	Passive + ligand-directed active targeting	Mostly passive; some active via surface chemistry	Passive; active via folate or antibody conjugates	Highly specific via native membrane proteins	Depends entirely on the chosen targeting ligands
